# Recent Advances in Nanomedicine for the Diagnosis and Treatment of Prostate Cancer Bone Metastasis

**DOI:** 10.3390/molecules26020384

**Published:** 2021-01-13

**Authors:** Daniel E. Hagaman, Jossana A. Damasco, Joy Vanessa D. Perez, Raniv D. Rojo, Marites P. Melancon

**Affiliations:** 1 Department of Interventional Radiology, The University of Texas MD Anderson Cancer Center, Houston, TX 77030, USA; dhagaman76@gmail.com (D.E.H.); jdamasco@mdanderson.org (J.A.D.); jdperez@mdanderson.org (J.V.D.P.); rdrojo@mdanderson.org (R.D.R.); 2 College of Medicine, University of the Philippines, Manila NCR 1000, Philippines; 3 UTHealth Graduate School of Biomedical Sciences, Houston, TX 77030, USA

**Keywords:** prostate cancer, bone metastasis, nanotechnology, nanomedicine

## Abstract

Patients with advanced prostate cancer can develop painful and debilitating bone metastases. Currently available interventions for prostate cancer bone metastases, including chemotherapy, bisphosphonates, and radiopharmaceuticals, are only palliative. They can relieve pain, reduce complications (e.g., bone fractures), and improve quality of life, but they do not significantly improve survival times. Therefore, additional strategies to enhance the diagnosis and treatment of prostate cancer bone metastases are needed. Nanotechnology is a versatile platform that has been used to increase the specificity and therapeutic efficacy of various treatments for prostate cancer bone metastases. In this review, we summarize preclinical research that utilizes nanotechnology to develop novel diagnostic imaging tools, translational models, and therapies to combat prostate cancer bone metastases.

## 1. Introduction

Prostate cancer (PCa) is the second most common cancer among men in the United States, accounting for 1 in 5 new diagnoses. It is estimated that there will be approximately 192,000 new cases and 33,000 PCa-related deaths expected in 2020 [1]. Surgical and hormonal therapies have shown beneficial effects only for early-stage, hormone-responsive disease. Improvements in screening and early interventions for PCa have allowed patients with localized and regional disease to achieve a 5-year survival rate of nearly 100% [1,2]. This number, however, drops significantly to around 30% among patients who develop distant metastases [3]. PCa cells are dependent on androgen stimulation [4]. Binding by androgens affect the transcription of androgen-regulated genes like prostate-specific antigen (PSA) and ultimately stimulates proliferation and inhibits apoptosis of malignant cell lineages in the prostate. Owing to this dependence, androgen-deprivation therapy by chemical and surgical castration has become an effective mainstay of treatment for early metastatic PCa. However, all patients invariably progress to become “castrate-resistant” or hormone-independent as the tumor adapts to the androgen-deprived environment. Among men receiving treatment for castration-resistant PCa, the bones are the most likely site of distant metastasis, with bone metastasis occurring in 80–90% of these patients [5,6]. Bone metastatic PCa commonly affects the long bones, pelvis, ribs, skull, and vertebral column [7]. Such a development is often indicative of poor prognosis and bone metastases often causing debilitating pain and impaired functioning that drastically reduce both quality of life and survivability [8]. Bone metastasis affects both bone structure and hematopoiesis. This results in significant morbidities for patients with advanced disease. Overgrowth and irregular bone formation can lead to pain, fractures, and compressive mass effect on surrounding tissues. Invasion and replacement of the bone marrow by metastatic PCa cells can lead to anemia and decreased immune activity [9]. A recent study demonstrated that anemia and thrombocytopenia due to the decline in bone marrow function results in significantly reduced survival times in PCa patients with skeletal lesions [10].

Bidirectional interactions between the tumor and the bone microenvironment are believed to drive the resulting pathophysiologic changes in PCa bone metastasis [11]. The bone matrix is composed primarily of Type I collagen comprising around 95% of the matrix. The remaining component is a mix of noncollagen proteins and proteoglycans including osteopontin, bone sialoprotein, and osteonectin [12]. Studies have shown these matrix components to be preferential for the attachment and growth of PCa cells [13,14]. The bone is also home to active cells in the bone comprised of osteoblasts, osteoclasts, hematopoietic cells, adipocytes, and a variety of immune cells. Dynamic cellular activity that regulate bone cell maturation facilitated by a host of growth factors combines with a favorable matrix to make a microenvironment suitable for metastatic tumor growth [15].

Bone metastasis is often described as one of two phenotypes defined by the activities of bone-forming osteoblasts (osteoblastic) and bone-lysing osteoclasts (osteolytic). Unlike osteolytic lesions caused by bone metastases from breast and other cancers, PCa uniquely induces bone formation [16]. An exception, albeit uncommon, is neuroendocrine tumors of the prostate which also produce osteolytic lesions [17].

Bone formation is a function of osteoblast activity. Osteoblasts embedded in the bone differentiate into osteocytes or undergo apoptosis if deposited to the new bone matrix. The newly formed bone matrix induced by osteoblasts is mineralized by the deposition of hydroxyapatite crystals [12]. PCa cells can alter bone homeostasis by secreting factors that either directly affect osteoblast functions or influence bone formation indirectly, by modifying the bone matrix or microenvironment [16]. Complex signaling pathways govern the growth and differentiation of osteoblasts in a process known as bone remodeling. Through the secretion of bone growth factors that activate osteoblasts, PCa cells stimulate bone formation in a manner that resembles the process involved in normal bone development and repair. This correlates with the observation of elevated bone formation and increased bone mineral density in PCa bone metastasis [18]. Despite this, tumor-generated bone tends to be abnormal and lack the typical lamellar bone structure of normal bone. As such, these tend to be weaker and more prone to fractures, which is a common clinical observation in PCa patients. The increased bone volume may, however, contribute to limiting tumor growth thus delaying progression of metastasis [19].

Evidence suggests that osteoblast themselves also contribute to the progression of PCa bone metastasis. Co-cultures of PCa cells with osteoblasts have been shown to have increased proliferation compared to those without osteoblasts [20]. Observation of this relationship has been suggested to occur only in PCa cell lines [21,22]. Osteoblasts are also noted to be able to regulate osteoclast activity through cytokines receptor activator of nuclear factor-κB ligand (RANKL), a key activator of osteoclast differentiation, and osteoprotegerin, a soluble decoy receptor that inhibits RANKL [23,24]. Elevation in serum osteoprotogerin has been observed in advanced cases of PCa [25,26]. Together, these findings indicate that osteoblasts function as the master switch for the progression of PCa in bone through the regulation of the proliferation of PCa cells and osteoclasts [16].

Furthermore, increased osteoblast activity in advanced PCa is supported further by elevated levels of serum alkaline phosphatase, a bone-specific biomarker, in patients with metastatic disease [26]. A clinical trial on zolendronic acid, a bisphonate osteoclast inhibitor, provides indirect evidence to the role of osteoblasts in the expansion of prostate bone metastasis. Results show that among patients with advanced PCa on the medication, bone loss was reversed but cancer progression was not slowed [27]. Meanwhile, clinical studies on bone-homing radiopharmaceuticals, which deposit preferentially at sites of increased osteoblast activity and bone synthesis, have shown improved survival and palliative benefits [28,29,30,31]. Further, clinical trials on atrasentan used as a receptor antagonist against entholin-1 (ET1), a mitogenic factor for osteoblasts, demonstrated inhibition of bone formation as well as anti-tumor activity [32]. These suggested osteoblast inhibition plays a direct effect on PCa progression [16].

Given the unique characteristics of bone metastatic PCa, this review discusses the role of nanotechnology as emerging applications in preclinical modeling, diagnosis, and therapy of PCa bone metastasis. Furthermore, this review examines potential avenues for investigation wherein nanomedicine can potentially exploit the osteoblastic nature of bone metastatic PCa.

## 2. Nanomedicine and Prostate Cancer Bone Metastasis

To be considered a nanomedicine, a therapeutic or diagnostic system (e.g., nanoparticle, liposome, micelle, etc.) should contain at least one component with dimensions on the scale of nanometers. More broadly defined, nanomedicine refers to the application of nanotechnology to the field of medicine [33]. Nanomedicine has led to advancements in the diagnosis and treatment of many types of cancer, including PCa bone metastasis [34,35]. Nanoparticle-based systems are advantageous because of the following properties: (1) multifunctionality, drugs and imaging agents may be incorporated; (2) multimodality, different imaging agents may be included to employ different imaging modalities; and (3) specific targeting, which can be achieved through passive and/or active targeting [36].

Passive targeting, including the enhanced permeability and retention (EPR) effect, as well as other ways in which nanoparticles accumulate in tumors, have been widely studied [37,38]. Understanding of the EPR effect has led researchers to design more effective nanoparticles that selectively accumulate in tumors based on their size. To be effective, passive targeting also requires long enough circulation times to reach the desired location and the ability to evade the body’s natural immune response to degrade and eliminate nanoparticles. The functionalization of a nanoparticle surface with poly(ethylene glycol) (PEG) or PEGylation, is a common strategy to achieve extended circulation times and evade the immune system.

**Figure 1:**
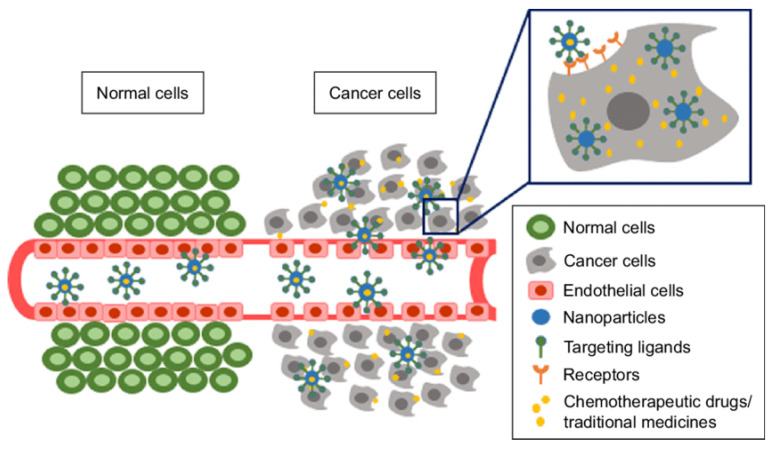
Nanoparticles can preferentially be taken up by cancer cells through passive targeting, active targeting (blue box), or both mechanisms. (Adapted from Ref. [40]).

Another way to increase nanoparticle concentration in a pre-determined location within the body is known as active targeting. This technique relies on functionalizing a nanoparticle with a ligand such as a small molecule, short polypeptide sequence, antibody, etc. that binds with high affinity and specificity to a receptor at the biological target of interest [39,40]. Both passive and active targeting strategies seek to take advantage of inherent differences between healthy cells and cancerous cells such as leaky vasculature of tumors (passive targeting) or the overexpression of proteins on the surface of cancer cells (active targeting) (Figure 1) [41]. Merging together technological advancements that have been made in this field have resulted in increasingly sophisticated and effective nanomedicines such as multimodal nanoparticles, which contain two different imaging contrast agents; multifunctional nanoparticles, which typically contain both an imaging contrast agent and a therapeutic agent; and nanoparticles with a wide range of targeting capabilities. Considering the possible combinations of material, size, shape, surface chemistry, stability, etc., the potential that nanoparticles have seems limitless, and therefore more breakthroughs in this field are expected. In this review, we discuss recent advances in preclinical research using nanomedicine to treat bone metastatic PCa, focusing on new strategies for improving diagnostic imaging and therapeutic management in translational models.

### 2.1. Preclinical Models

#### 2.1.1. In Vitro Bone Metastatic PCa Models

Cell lines of human origin that are frequently used in bone metastatic PCa models include PC3, LNCaP, DU145 and the sublines derived from them [16,42]. Non-human cell lines used in bone metastatic PCa models include the rat-derived R-3327 line [43,44], which forms osteoblastic lesions similar to those observed in humans, and the canine-derived ACE-1 cell line, whose intratibial or intracardiac injection gives rise to bone metastases [42,45]. In addition, patient-derived xenograft MDA-PCa-118b (PCa-118b) possesses osteogenic potential that can induce bone formation when implanted in mouse femur and also when inoculated subcutaneously [46]. Most recently, Lin et al. showed that bone morphogenetic protein (BMP)- 4 secreted by PCa cells converts tumor-associated endothelial cells into osteoblasts, which represent one of the mechanisms by which osteoblastic lesions of bone metastatic PCa are formed. Furthermore, they have shown that overexpression of BMP4 in non-osteogenic C4-2b PCa cells induced ectopic bone formation when implanted subcutaneously [47].

Most in vitro experiments are traditionally carried out in 2-dimensional flat-bottom cell culture plates, although in some instances they are not representative of in vivo cell growth [48,49]. Compared with traditional, 2-dimensional cell cultures, 3-dimensional (3D) cell cultures have more cell–cell and cell–matrix interactions and thus more closely resemble physiological conditions [50]. To create a better simulation of the bone microenvironment for use in experiments with PCa cells, Fitzgerald et al. developed a 3D collagen scaffold containing nanoscale hydroxyapatite crystals for cell cultures [51]. To mimic the interactions between cancer cells and bone tissue cells, the authors co-cultured either PC3 or LNCaP cells with human fetal osteoblasts (hFOB 1.19 cells). Compared with the PCa cells cultured alone, those co-cultured with the osteoblasts had decreased proliferation and increased expression of the MMP9 enzyme, a biomarker of PCa metastasis [52,53]. The authors were also able to use a cyclodextrin-based nanoparticle delivery vector to knock down the *RelA* gene in their 3D co-culture. RelA is a subunit of the NF-κB transcription factor and is involved in the metastatic cascade of PCa [54]. Improved in vitro models that more accurately predict in vivo behavior are vital to the development of the next generation of bone metastatic PCa treatments. Another 3D in vitro platform to study the progression of PCa and its adaptive response in bone microenvironment was developed by Bock et al. [55]. Here, they bioengineered a human osteoblast-derived mineralized microtissue (hOBMT) by seeding human primary osteoprogenitor cells on a calcium phosphate-coated 3D printed scaffold consist of medical-grade poly(caprolactone). Osteogenic differentiation of hOBMT resulted to a human osteoblast-type organization with highly viable and dense extracellular matrix/collagen-type fibrils deposition, with osteoblastic and osteocytic morphologies, that partly display marker profile of osteocytogenesis lacking in 2D models. When co-cultured with PCa cells (i.e., androgen receptor (AR)-positive and dependent LNCaP, AR-positive and independent bone metastatic C4-2B, and AR-negative bone metastatic PC3) in an androgen-deprived condition, PC3 showed attachment unaffected by medium, while both LNCaP and C4-2B had increased attachment than in the androgen-replete condition, however, only C4-2B showed significant (*p* < 0.001) increased attachment to bone, indicating acquisition of androgen-independent features. No morphological differences were observed for C4-2B under androgen deprivation, while LNCaP displayed highly elongated morphologies typical of epithelial-to-mesenchymal transition or neuroendocrine transdifferentiation, which are established adaptive response of PCa to escape androgen deprivation, which could signal transition to the more aggressive castrate resistance phenotype. The ability of hOBMT to reproduce cellular alterations observed in vivo with androgen deprivation offers a promising approach for discovery and testing of relevant biomarkers and therapeutics for osteoblastic PCa.

#### 2.1.2. In Vivo Bone Metastatic PCa Models

Recreating every aspect of bone metastatic PCa progression in an animal model has yet to be achieved [56]. Preclinical studies typically utilize models that replicate only a portion of the overall disease. One common method of creating bone metastatic PCa models involves injecting cancer cells into the tibias of mice. This procedure is relatively easy, yields a high rate of tumor formation within the bone, and enables the study of cancer cells in the bone environment [57]. However, bone metastatic PCa models created this way do not recreate other events associated with the metastatic cascade. Another method used to create bone metastatic PCa models involves injecting cancer cells into the left ventricles of the hearts of mice. This method is more technically challenging than other inoculation methods, and its rate of tumor formation varies greatly depending on the cell lines used [58]. Compared with those in models created by intraosseous injection, the bone metastases that form in models created with intracardiac injection are more pathologically similar to human bone metastases, as these models mimic some aspects of the naturally occurring metastatic cascade [59]. Depending on the cell line, bone metastatic PCa models created with intracardiac injection can form osteolytic or osteoblastic lesions. Commonly used human-derived cell lines that result in osteolytic lesions include DU145, PC3, and PC3M-LN4 [57,60]. LNCaP and its sublines (LNCaP C4-2 and LNCaP C4-2B) cause osteoblastic lesions [61].

Bone implant models are also used to study PCa in the bone microenvironment [62,63]. Unlike intraosseous models, bone implant models enable researchers to study the interactions between human cells lines and human bone or human bone tissue that have been implanted subcutaneously [64,65,66]. Recently, Landgraf et al. were able to replicate the therapeutic effects of zolendronic acid (ZA) in a humanized bone implant model in male NSG mice [67]. They created a human tissue-engineered bone construct (hTEBC) that consisted of a hydrogel seeded with human osteoblasts, umbilical vein endothelial cells, and multipotent mesenchymal stromal cells wrapped with medical grade polycaprolactone. Twelve weeks after the subcutaneous implantation of the hTEBC, the intracardiac injection of PC3-Luc cells initiated the growth of PCa metastases. Ex vivo bioluminescence imaging and tartrate-resistant acid phosphatase-positive tissue staining revealed that the PCa cells grew preferentially on the hTEBC. In addition, ex vivo imaging demonstrated that the ZA treatment group had a smaller metastatic load than the control group did until week 16 (*p* < 0.01). Immunohistochemical staining for human CD44 revealed that the ZA group had significantly fewer PCa-luc cells in the liver than the control group did (*p* = 0.020). Although the model does not recapitulate every aspect of bone metastatic PCa, its microenvironment is biologically relevant for studying the therapeutic effects of ZA on BMPCa.

### 2.2. Diagnostics

#### 2.2.1. Current Clinical Diagnostic Modalities

The early diagnosis of skeletal-related events is crucial to improving the outcomes of PCa patients. Imaging techniques such as bone radiography, bone scintigraphy, whole-body magnetic resonance imaging (MRI), positron emission tomography (PET)-computed tomography (CT), and image-guided biopsy can be used to determine if a skeletal lesion is due to metastatic disease [68]. Several factors determine how these diagnostic tools are implemented throughout the treatment of PCa. State-of-the-art diagnostic imaging tools for the assessment of metastatic bone disease are described in detail elsewhere [69]. Here, we briefly discuss diagnostic imaging research related to bone metastatic PCa.

Bone scintigraphy techniques such as single-photon emission CT have high sensitivity for bone lesions because the radiotracers developed for the procedure (e.g., ^99m^Tc-3,3-diphosphono-1,2-propanodicarboxylic acid) accumulate in remodeling bone [70,71]. However, single-photon emission CT may not easily distinguish between metastatic bone disease and a benign skeletal defect such as an osteoarthritic lesion. The limitations of bone scintigraphy have sparked interest in PET imaging in bone metastatic PCa. ^18^F-fluorodeoxyglucose (18FDG) is an analogue of glucose that reflects increased glucose metabolism associated with several tumor types [72]. On the other hand, ^18^F-fluoride is taken up by bone metastases and related to their osteoblastic activity and shows a high contrast between normal and abnormal bone due to higher turnover of abnormal osteoblasts [73]. Other PET tracers being evaluated include ^11^C-methionine and ^11^C/^18^F-choline and ^11^C-acetate derivatives. Due to higher cell turnover, malignant cells have elevated levels of choline which is used for the synthesis of phospholipids [73]. ^11^C-choline PET is advantageous over ^18^F-FDG PET for detection of pelvic disease and bone metastases because urinary excretion is negligible. However, ^11^C-choline accumulates in liver, kidney, spleen and pancreas, making assessment of the upper abdomen difficult [74]. More recently, the expression of the prostate-specific membrane antigen (PSMA) was targeted with ^68^Ga-labeled PSMA ligands [75,76]. ^68^Ga-PSMA-11 PET/CT assists in disease localization at biochemical recurrence (BCR), staging, and to select patients for PSMA-targeted molecular radiotherapy [75].

Diffuse-weighted MRI (DW-MRI) can quantitatively assess the Brownian (i.e., random) motion of water molecules in tissue [77,78]. Conventional MRI scans do not contain information about the rate of water diffusion through tissue; thus, DW-MRI is a more sensitive technique for estimating the cellular density of a region of interest [79] and is well-suited for oncology applications, since water diffuses through healthy and cancerous tissues differently. From DW-MRI scans, an apparent diffusion coefficient (ADC) can be calculated and used to describe a region of interest (e.g., a suspected tumor). Changes in the ADC in response to disease progression or treatment describe both size and density/cellularity of the tumor which can give valuable insight to clinicians [80,81]. One active area of research in this field is the development of new algorithms that create predictive models to improve diagnostic sensitivity/specificity [82] or enhance treatment response data [83,84,85,86].

**Table 1:** Nanoparticles developed for prostate cancer (PCa) bone metastasis diagnostics.

Name	Core Material	Surface Functionalization	Imaging Modality	Current Status	Refs
Ferumoxtran-10	SPIO	Dextran	MRI	Phase 3 Clinical Trial—Europe Ferrotran^®^	[87]
Ferumoxytol	SPIO	Carboxymethyl dextran	MRI	FDA approved (2018) for iron deficiency anemia	[88]
GNPs	Gold	Aptamer	CT	Pre-Clinical	[89]
Immunolabled QDs	ZnS capped CdSe	Antibody	Optical	Commercially available antibody labeling kits	[90,91]
Radiolabled IONPs	SPIO	RGD peptide, Poly(aspartic acid), Radionuclide chelator	PET and MRI	Pre-Clinical Investigation	[92]
Radiolabled QDs	[^64^Cu]CuInS/ZnS	PEG	PET and Optical	Pre-Clinical Investigation	[93]
IONPs/QDs	Liposomal SPIO and CdSe QDs	PEG, RGD peptide	MRI and Optical	Pre-Clinical Investigation	[94]
C’ dots	Amorphous Silica (SiO_2_)	PEG, PSMA targeting peptide, radionuclide chelator	PET and Optical	Phase 1—Guided Surgical Treatment of Prostate Cancer Phase 1—Head and Neck Melanoma; Phase 2—Colorectal Cancer	[95]

Abbreviations: SPIO, superparamagnetic iron oxide; MRI, magnetic resonance imaging; QDs, quantum dots; GNPs, gold nanoparticles; CT, computed tomography; IONPs, iron oxide nanoparticles; RGD, arginylglycylaspartic acid; PET, positron emission tomography; PSMA, prostrate specific membrane antigen; PEG, poly(ethylene glycol).

#### 2.2.2. Nanoparticles for Bone Metastatic PCa Diagnostics

Accurate staging for those diagnosed with PCa is important to clinicians so they can provide the most appropriate treatments especially for more advanced disease with metastatic involvement [95]. Metastatic adenocarcinoma in male patients is routinely assessed for prostatic origin by using prostate-specific antigen (PSA) [96]. However, the utility of PSA is limited since benign prostatic lesions and some non-prostatic tissues also express PSA in addition to a loss in PSA expression in distant metastases by 10–20% [97]. Additional markers for primary prostatic cancers have been investigated, including prostate-specific membrane antigen (PSMA), prostate-specific acid phosphatase (PSAP), prostein (also known as p501s or SLC45A3), ETS-related gene (*ERG*), androgen receptor (AR) [98,99,100,101,102,103,104]. Queiser et al. have investigated the expression of these markers for lymph nodes and distant metastases, primarily of osseous origin, and found that all markers except ERG were able to be detected in lymph node and distant metastases. PSA, PSMA, and AR had the highest sensitivity (97%, 94%, and 91%, respectively) and this can be further increased to 98–100% by combining PSA with PSMA or AR [105].

In this section, the diagnostic utility of nanoparticles in imaging and immunohistochemical analyses are discussed, as well as their use in targeted imaging for PCa and PCa metastasis. The nanomaterials discussed above are listed in Table 1.

A. MRI

Superparamagnetic iron oxide (SPIO) nanoparticles have been investigated as MRI contrast agents for the evaluation of lymph nodes for various forms of cancer, including PCa [106]. Ferumoxtran-10 and Ferumoxytol are two closely related SPIO nanoparticles developed for this application. Ferumoxtran-10 consists of 5 nm iron oxide nanoparticles coated with the natural polysaccharide dextran. Dextran polymer chains consist of glucose molecules connected through α-1,6 glycosidic linkages with varying degrees of branching between the α-1,3 positions. Dextran binds to the surface of the SPIO, creating a stable aqueous dispersion and also prolonging the circulatory time of the nanoparticles [107]. A phase 2 dosing study for Ferumoxtran-10 demonstrated consistent strong enhancement (T2/T2* weighted) of normal nodes at 24- and 36-h timepoints for both 2.4 and 3.6 mg Fe/kg doses [108]. Despite initial promising results, Ferumoxtran-10 was never granted FDA approval for reticuloendothelial MRI imaging.

Ferumoxytol uses a similar SPIO nanoparticle core as Ferumoxtran-10 and was also developed for MRI imaging applications, however its iron oxide core is coated with the semi-synthetic polymer carboxymethyl dextran (CMD). CMD is a derivative of dextran where a fraction of the hydroxyl (R-OH) functional groups of the glucose subunits have been replaced with carboxymethyl groups (R-CH_2_COO-Na^+^). The ionic nature of the carboxymethyl groups of CMD enhances its water solubility and binding to the surface of the iron oxide nanoparticle [109]. A recent Phase I dosing study for MR lymphography in patients with PCa found a homogenous and dose-dependent loss in signal intensity for normal lymph nodes at a 24-h time point and 7.5 mg Fe/kg concentration [87]. Due to insufficient signal loss, lower doses than 7.5 mg Fe/kg could lead to high false-positive rates when trying to determine benign lymph nodes from malignant ones. When compared to Ferumoxtran-10, the authors concluded that higher doses of Ferumoxytol were needed due to the shorter half-life (25–30 h vs. 15 h) and less uptake from macrophages due to differences in the polysaccharide coatings [87]. While no significant adverse events were reported from this dosing study, further studies would need to determine the efficacy of Ferumoxytol as an MRI contrast agent for this application.

B. Computed tomography

Gold nanoparticles (AuNPs) are one of the most extensively studied nanotechnology platforms with applications in various fields such as electronics [110], sensors [111], chemical catalysis [112], therapeutics [113], and diagnostics [114]. Kim et al. have developed a AuNP that actively targets the prostate specific membrane antigen (PSMA), a protein overexpressed on the surface of PCa cells. Using thiol chemistry, they conjugated an RNA aptamer to the surface of AuNP that binds to PSMA with high affinity and specificity. Like previously reported AuNPs modified with targeting ligands the authors demonstrated their AuNPs could be used as a CT contrast agent [88]. Using a clinical CT instrument, they imaged cell suspensions that had been previously incubated with the AuNPs. LNCaP cells (PSMA+) showed a 4-fold increase in signal intensity (Hounsfield Units) when compared to PC3 cells, which did not express PSMA. Other experiments such as silver staining and inductively coupled plasma atomic emission spectroscopy (ICP-AES) confirmed that the AuNPs were binding selectively to cells expressing PSMA. CT is a widely used diagnostic imaging tool, so the development of CT contrast agents with high specificity for relevant biological targets are of great interest.

C. Optical

Immunohistochemistry (IHC) is commonly used to analyze the tissue from biopsy samples. The results can provide important information for both the diagnosis and treatment plan. Many IHC protocols employ fluorescent probes where the fluorophore is typically an organic dye (e.g., rhodamine). Quantum dots (QDs) have several advantages over organic dyes or fluorescent proteins such as narrow and symmetric emission spectra, resistance to photobleaching, superior brightness, and tunable emission [115]. Ruan et al. compared conventional IHC to a QDs-based immunolabeling technique in detecting the expression of prostate stem cell antigen (PSCA) in human prostate tissue. Studies have shown that PSCA has prognostic utility since its expression correlates with tumor stage, grade, and androgen independence [89,90]. Although not statistically significant, the QDs-based assay had a slightly higher positive PSCA expression rate (76%, 61/80) than the conventional IHC rate (70%, 56/80). Notably, the intensity of the fluorescent signal from the QDs specimens remained stable for two weeks, and approximately 93% of the specimens with positive expression could be detected after four weeks. While the prolonged photostability of the QDs-based probe is a positive attribute, this technology may not be widely used if clear sensitivity or specificity advantages are demonstrated. In addition, other factors such as cost and environmental impact should be considered when comparing new technology to existing standards.

D. Combination/Multimodal

The development of bifunctional imaging probes with oncological applications is also an active area of bone metastatic PCa research. Theoretically, two or more imaging techniques could be combined to offset the weaknesses of either imaging technique alone. This multimodal approach is made possible with nanomaterials, which can be loaded with different reporters and delivered to specific target areas. For example, Lee et al. developed PET/MRI multimodal nanoparticles by binding the radionuclide ^64^Cu-1,4,7,10-tetraazacyclododecane-1,4,7,10-tetraacetic acid chelate, a PET contrast agent, to the surface of poly(aspartic acid)-capped iron oxide nanoparticles, which serve as an MRI contrast agent. The surface of these nanoparticles was also functionalized with the cyclic arginylglycylaspartic acid (RGD) peptide, which binds to integrins overexpressed by cancer cells, yielding a multimodal nanoparticle that had enhanced uptake in tumor cells and could generate contrast for both PET and MRI [91]. PET/optical imaging multimodal nanoparticles were also demonstrated by Guo et al. The authors made these quantum dots intrinsically radioactive by using ^64^CuCl_2_ as the synthetic precursor to CuInS/ZnS hybrid nanoparticles [92]. The PEGylation of these nanoparticles increased their tumor uptake, and the particles generated contrast for PET and, owing to their photoluminescence, could be optically imaged at an emission wavelength of approximately 700 nm. Although these examples of multimodal nanoparticles were designed for general cancer imaging applications, other researchers have designed multimodal imaging agents for the diagnosis of specific diseases, such as bone metastatic PCa.

Wang et al. synthesized liposomes that contained both magnetic and fluorescent contrast agents for MRI and optical imaging, respectively [93]. The pairing of the imaging modalities is logical; in vivo, fluorescence imaging has high sensitivity but poor anatomical resolution, whereas MRI has excellent anatomical resolution but occasionally lacks sensitivity [116]. Within their liposomes, Wang et al. encapsulated dimercaptosuccinic acid-capped iron oxide nanoparticles, which are hydrophilic and can be dispersed in water, as the MRI contrast agent [117]. The fluorescent nanoparticles they used were CdSe quantum dots capped with oleic acid, which are hydrophobic. They conjugated a cyclic RGD cancer cell-targeting peptide to the surface of the liposomes to increase their tumor uptake [118]. They then tested their liposomes using a bone metastatic PCa mouse model established by injecting mouse RM-1 PCa cells into the tibias of C57BL/6 mice. Both MRI and fluorescence imaging revealed that after 4 h, the RGD-modified nanoparticles accumulated in the tumor, which could be distinguished from surrounding healthy tissue. In contrast, the nanoparticles without the RGD peptide were mainly taken up by the liver, and the signal-to-noise ratio was not high enough to clearly distinguish the tumor on either MRI or fluorescence imaging.

Researchers at Cornell university have developed ultra-small amorphous silica nanoparticles with both diagnostic and therapeutic properties for cancer management. These nanoparticles, which they have given the moniker Cornell Prime Dots or C′ dots, are renally clearable due to their small size (6–7 nm diameter) and have multiple functionalities. Using functionalized silane precursors, C′ dots have an amorphous silica core that encapsulates a fluorescent dye (Cyanine dyes, Cy5) for optical imaging, as well incorporates PEG surface groups and reactive functional groups for chemical derivatization in subsequent synthetic steps [94]. To use C′ dots for both targeting PCa tumors and PET imaging, the authors derivatized the surface of the nanoparticles with a peptide that binds to PSMA and deferoxamine (DFO), which can chelate radionuclides (e.g., ^89^Zr, ^64^Cu, ^68^Ga). The authors tested their PCa targeting C’ dots in a mouse model (male NOD SCID, 6–8 weeks old) using tumor xenografts established by injection of LNCaP (PSMA+) and PC3 (PSMA-) cells subcutaneously. Significant differences (** *p* < 0.005) in tumor uptake was observed by PET imaging at all timepoints (24, 48, and 72 h) between the LNCaP and PC3 models, with maximum nanoparticle uptake occurring at 48 h. Biodistribution studies support the PET imaging results that the LNCaP group saw a 2-fold higher tumor uptake in C′ dots, which shows that these nanoparticles can selectively bind to PCa (PSMA+) cells in vivo. These results demonstrate the versatility of the C′ dots platform and also highlight how both size and surface functionality play a role in nanoparticle performance. This technology is currently being evaluated in a Phase 1 clinical trial to determine if it is a safe and effective way to identify tumor cells during prostate cancer surgery [119].

### 2.3. Therapeutics

#### 2.3.1. Current Therapies for Bone Metastatic PCa

The current standard of care for patients with metastatic bone pain comprise appropriate anti-tumor management via radiotherapy combined with early initiation of bone-targeting agents (e.g., bisphosphonates, denosumab) and adequate use of opioid and non-opioid analgesics. Second generation anti-androgen drugs such as enzalutamide and abiraterone have recently been approved for castration resistant PCa. They are typically indicated for use after patients no longer respond to initial hormonal therapies or treatment with docetaxel. Both drugs have demonstrated significant overall survival benefits and as such they have become standard treatments for those with castrastion resistant PCa [120]. In addition, surgical management through excision or even amputation is sometimes indicated for complicated cases of metastatic bone disease [8]. Existing treatments for bone metastatic PCa are discussed in detail elsewhere [121]. Here, we briefly summarize the four bone metastatic PCa treatments related to the clinical and pre-clinical research covered in this review: (1) radiopharmaceuticals, (2) bisphosphonates, (3) chemotherapy, and (4) targeted therapy, such as denosumab.

A. Radiopharmaceuticals

Radionuclides such as strontium-89 and samarium-153 are β-emitters that can accumulate in bony metastatic lesions and locally release ionizing radiation. These agents rapidly reduce pain but do not increase overall survival times [29,122,123]. Radium-223 dichloride (trade name Xofigo) is an α-emitter that can act as a calcium mimic and accumulate in remodeling bone. An initial study in bone metastatic PCa patients found this treatment to not only relieve pain [124,125] but also delay prostate-specific antigen level progression, normalize alkaline phosphatase levels, and extend overall survival times by several months. These benefits were accentuated in patients also taking bone supportive agents such as denosumab or bisphosphonates. After a data review of the Phase 3 ERA-223 clinical trial in 2018 the European Medicines Agency restricted the use of radium-223 dichloride in combination with abiraterone acetate and prednisone or prednisolone over concerns that combining these drugs increases the risk of fractures and might reduce overall survival times [126]. Further studies are needed since combining Radium-223 with current treatments for metastatic PCa such as bone supportive agents, androgen blockers, or immunotherapies will likely provide therapeutic benefits. Currently, there are five phase 2/3 clinical trials ongoing that are evaluating these drug combinations (ClinicalTrials.gov: NCT02346526, NCT02463799, NCT02225704, NCT01929655, NCT02194842) [127].

B. Bisphosphonates

Bisphosphonates, a class of synthetic drugs that mimic pyrophosphate, a naturally occurring component of bone, strongly bind to hydroxyapatite crystals on the bone surface and cannot be easily removed by hydrolysis [128,129]. Bisphosphonates inhibit osteoclast activity by interrupting the biosynthesis of farnesyl pyrophosphate [130]. They have been shown to reduce skeletal-related events, reduce the risk of fracture, and preserve bone mineral density [131,132]. A promising strategy for the development of bone-targeting nanoparticles is to conjugate amino bisphosphonates (e.g., alendronate, pamidronate) to the surface of nanoparticles [133,134]. The enhanced binding of the nanoparticles to the bone increases the likelihood of delivering the nanoparticle payload to tissue affected by bone metastatic PCa.

C. Chemotherapeutics

Taxane-based chemotherapy is commonly used to treat bone metastatic PCa. The first chemotherapy drug to improve the survival of men with advanced PCa was docetaxel (DTX) [135,136], which was also shown to decrease pain, improve quality of life, and reduce serum levels of prostate-specific antigen. For patients whose PCa progresses during or after DTX treatment, the U.S. Food and Drug Administration has approved cabizataxel (CBZ), which has demonstrated survival benefits [137,138]. The drawbacks of these treatments include the development of drug resistance and toxicity-related side effects. Developing nanosized drug delivery systems that overcome these and other issues is the focus of substantial preclinical research.

D. Denosumab

Denosumab is a fully human monoclonal antibody that interrupts RANK-RANKL signaling. It binds RANKL with high affinity, preventing the natural ligand from binding to RANK [139]. This reduces both mature osteoclast activity and osteoclast differentiation, which restores the balance between the cellular functions of osteoblasts and osteoclasts. In 2011, the results of a phase III clinical trial of denosumab in patients with castration-resistant PCa were published. Denosumab treatment prolonged bone metastasis-free survival (*p* = 0.028) and increased the time to first bone metastasis (*p* = 0.03) [140]. When compared to traditional cancer treatments such as radiation therapy or chemotherapy, monoclonal antibodies represent a paradigm shift in cancer treatment. They have high specificity for their biological targets (e.g., cancer cells [141], PD-1 [142], PD-L1 [143], CTLA4 [144]), have reduced systemic toxicity, and elicit sustained anti-tumor responses.

Current bone metastatic PCa treatments, such as bisphosphonates (e.g., ZA; trade name Zometa), work by restoring bone homeostasis [145]. Such treatment is only palliative; although it can strengthen bone and prevent fractures, it does not treat the underlying cancer or significantly extend patient survival [146]. Therefore, curative treatments for bone metastatic PCa are urgently needed.

#### 2.3.2. Nanotechnology-Based Bone Metastatic PCa Therapy

Many cancer drugs are lipophilic and therefore must be formulated into a stable emulsion or suitable drug delivery platform before they can be administered to patients. Ideally, these formulations should reach their target location without being degraded, release the drug at an appropriate rate, and be inherently biocompatible. Encapsulating cancer drugs within liposomes [147,148], polymeric nanoparticles [149], or other drug delivery vehicles can provide a stable environment for a hydrophobic drug in an aqueous environment. Liposomes and polymeric nanoparticles provide a physical barrier between the drug and biological fluids that can quickly degrade cancer drugs that have sensitive functional groups. These nanoparticles release their contents when they are degraded, typically described as burst release as the majority of encapsulated content is expelled at this point [150]. If the nanoparticle is not at the desired location when they are degraded, then the majority of the encapsulated drug will not be delivered to the intended target [151]. Polymer-drug conjugates can also solubilize lipophilic drugs and in some instances encapsulate the drug, although this depends on the exact properties of the conjugate such as molecular weight, water solubility, concentration, etc. [152,153]. Release of the biologically active compound from conjugates depends on the type of bond between the polymer and drug (i.e., ester, amide, hydrazone) and any steric hinderance the polymer causes to slow the cleavage of the bond. In the following sections we highlight how nanoparticles such as liposomes, polymeric nanocapsules, and polymer-drug conjugates are being used to fight bone metastatic PCa. As discussed previously in Section 2.3.1, there are multiple treatment options available to patients with advanced PCa. Current research efforts with nanomedicine aim to overcome limitations of current treatments such as dose limiting toxicity, drug resistance, and generally provide more effective therapeutics. Here, we discuss some of the preclinical research aimed at developing such treatments and provide a list of treatments in Table 2, organized by drug delivery vehicle, material, and therapeutic agent.

A. Liposomes

When dispersed in water, phospholipids spontaneously form liposomes which consist of a lipid bilayer that separates an internal aqueous core from the surrounding bulk aqueous phase [164]. The diameter of liposome particles can range from approximately 25 nm to 2.5 µm, however injectable liposomal formulations developed thus far for clinical use are less than 200 nm [165]. Liposomes are capable of encapsulating both hydrophobic and hydrophilic drugs. Hydrophobic drugs are incorporated into the lipid membrane of the liposome, stabilized by non-covalent interactions between the non-polar fatty acid segments of the phospholipid. When hydrophilic drugs are encapsulated by liposomes, they are carried within the aqueous core. Liposomes with active targeting properties have been demonstrated by chemically modifying phospholipids with ligands such as peptides, proteins, antibodies, and aptamers [166]. Depending on the size and size distribution of the liposomes, they can also passively target the tumor microenvironment by taking advantage of the EPR effect. These technological developments make the liposomal drug delivery platform a powerful tool for creating the next generation of cancer treatments.

Disintegrins, a family of small, non-enzymatic proteins found in snake venom, can bind to and modulate the function of human integrins [167,168]. Vicrostatin (VCN) is an engineered recombinant RGD disintegrin consisting of a single polypeptide chain of 69 amino acids; its native form, contortrostatin, is a component of snake venom [169]. Disintegrins have high affinity and specificity for activated conformations of surface integrins on motile cells (e.g., tumor cells, angiogenic endothelial cells) [170]. VCN disrupts the integrin-mediated signaling pathways that cancer cells use, including the PI3K, Src, MAPK, and FAK pathways, thereby decreasing both metastatic potential and overall survival of the cells [170]. Swenson et al. performed in vivo studies of VCN using a bone metastatic PCa model they created by injecting androgen-dependent CWR22rV1 cells into the tibias of athymic nude mice [162]. The tumors grew for approximately 3 weeks, when intravenous injections of 100 ug-equivalent doses of VCN were administered two times per week for 5 weeks [162]. The treatment groups received saline (control), empty liposome, naked VCN peptide, or liposomal VCN. Only the liposomal VCN group had a significant inhibition of metastatic tumor growth. The liposomal formulation, which benefits from the enhanced permeability and retention effect, also protects VCN from degradation. The liposomal formulation enables the native form of VCN to accumulate within the tumor, enhancing its efficacy.

B. Polymeric Nanoparticles

Aliphatic poly(esters) are one of the most widely used polymers for drug delivery applications since they have exceptional biodegradable and biocompatible qualities. Poly(lactic acid), poly(glycolic acid), poly(ε-caprolactone), and poly(lactide-co-glycolide) (PLGA) are the most frequently employed. Out of those polymers, PLGA stands out for the fact that it is approved by the Food and Drug Administration for drug delivery. The major degradation products of PLGA are lactic acid and glycolic acid which pose minimal toxicity risks. These chemicals are normal by-products of various metabolic pathways, however high enough local concentrations of the acidic degradation products have been shown to cause tissue inflammation adjacent to the implanted biomedical device [171]. Using the oil-in-water emulsion technique, PLGA nanoparticles that encapsulate hydrophobic or hydrophilic molecules can be prepared [172].

Adjei et al. developed nanoparticles that consist of PLGA, poly(vinyl alcohol), and the cationic surfactant cetyltrimethylammonium bromide. The authors varied the relative ratios of these components to yield nanoparticles with neutral, positive, or negative surface charges. The diameters of the nanoparticles were approximately 150 nm, slightly smaller than the intracellular fenestrations of bone marrow sinusoidal capillary endothelial cells [173]. The authors encapsulated paclitaxel (PTX) in these nanoparticles and then investigated their efficacy against bone metastatic PCa [154]. They created a bone metastatic PCa model by injecting PC-3M-luc cells into the tibias of male athymic nude mice. Tumor progression was monitored by assessing changes in the bioluminescence signal intensity, and in vivo fluorescence imaging was used to determine the biodistribution of the nanoparticles over time. Compared with the positively or negatively charged particles, the neutral nanoparticles showed 2.5-fold higher accumulation in the tibia after 96 h. Flow cytometry confirmed that most nanoparticles were located in the bone marrow, rather than on the bone surface. Compared with the control (saline) and a PTX ChremophorEL formulation (PTX-CrEL), neutral PTX nanoparticles slowed tumor growth 5 weeks. Micro-CT revealed significant bone deterioration and fractures in the control and PTX-CrEL treatment groups but only minor bone loss in the neutral PTX nanoparticle group. These experiments show how the passive targeting of nanoparticles can improve the efficacy of chemotherapy drugs by increasing their local concentration in the tissue.

**Figure 2:**
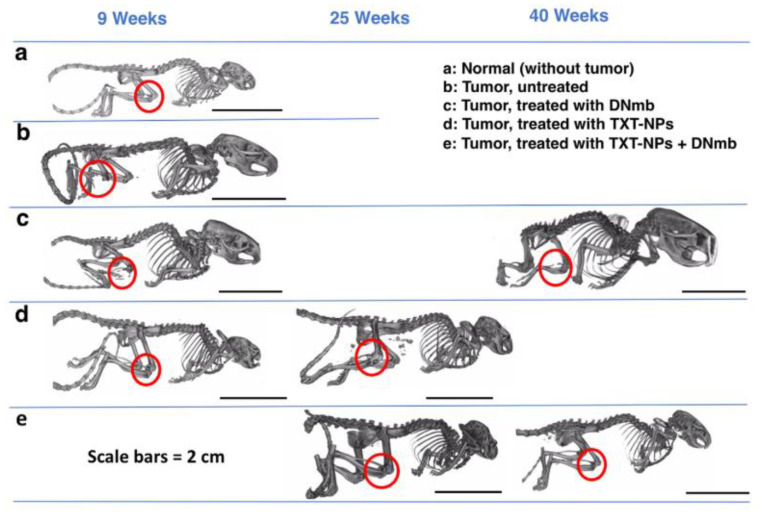
Micro-CT analysis for bone loss. Representative animals from different treatment groups underwent microCT at 9, 25, and 40 weeks. Group **a**: Normal (without tumor), **b**: Tumor, untreated, **c**: Tumor, treated with DNmb, **d**: Tumor, treated with TXT-NPs, **e**: Tumor, treated with TXT-NPs & DNmb. The combination treatment inhibited bone resorption most effectively. Histochemical analyses of the bone of the combination-treated animal demonstrated normal bone morphology, alkaline phosphate activity (a marker of osteoblasts), and tartrate-resistant acid phosphatase activity (a marker of osteoclasts). Reproduced with permission from [155].

Two agents commonly used to treat bone metastatic PCa are DTX and denosumab. Their mechanisms of action are different; whereas DTX is an antineoplastic agent, denosumab is a human monoclonal antibody that binds to RANKL, thereby inhibiting osteoclast activity [174]. Vijayaraghavalu et al., using an intraosseous model with PC3-luc cells in athymic nude mice, investigated whether delivering these two agents simultaneously with nanoparticles would be more effective than giving either agent alone [155]. The authors encapsulated DTX in poly(d,l-lactide-co-glycolide) nanoparticles, which they administered intravenously. The particles demonstrated a sustained release profile, delivering approximately 75% of the DTX over 7 days. Owing to the size of the nanoparticles, they passively targeted bone tissue [175], and their concentration in bone metastases was 3-fold higher than that of the CrEL-DTX formulations 1 week after administration. Mice treated with either DTX nanoparticles or denosumab initially showed an improved tumor response, but the tumors progressed after the treatments were stopped. The combination of DTX nanoparticles and denosumab showed no bone loss (Figure 2), and the tumor did not relapse after the treatment was stopped. Vijayaraghavalu et al. attributed these results to the nanoparticles’ slow release of DTX, which accumulated within the bone to halt cancer progression, and denosumab’s contribution to restore bone homeostasis. It would be interesting to see if DTX and other bone metastatic PCa treatments, such as bisphosphonates, would have similar synergistic effects in the model Vijayaraghavalu et al. used.

C. Polymer-Drug Conjugates

Although not currently used for the treatment of bone metastatic PCa, albumin-bound PTX (trade name Abraxane) is an example of a clinically successful polymer-drug conjugate used to treat multiple forms of cancer such as breast, lung, and ovarian cancers. In some instances, this PTX conjugate has higher efficacy and lower toxicity than traditional PTX formulations (e.g., Cremophor EL) [176]. In addition, unlike some nanoparticle drug delivery vehicles, Abraxane has a linear pharmacokinetic profile, making plasma concentration predictions accurate for clinically relevant dosages [177]. Pharmacokinetic profiles can be tuned with drug conjugates by altering the strength of the bond or making the bond pH-sensitive or redox-active [178,179,180]. The versatility of polymer-drug conjugates has made them the subject of intense research for many illnesses and here we describe several studies using polymer-drug conjugates to treat bone metastatic PCa.

Hoang et al. [158] developed nanoparticles consisting of Cellax polymers, poly(ethylene glycol) (PEG)-modified carboxymethylcellulose (CMC), and DTX. The authors used carbodiimide coupling chemistry to attach both PEG and DTX to the CMC polymer backbone through covalent ester bonds [181]. Because Cellax is an amphiphilic polymer and DTX is hydrophobic, nanoparticles with a DTX core and CMC shell stabilized by PEG can be prepared by controlled precipitation into aqueous solutions. Hydrophilic PEG chains extend from the surface of the nanoparticle in an aqueous environment to create a steric barrier that reduces the nanoparticle’s interactions with other particles [182]. To evaluate the efficacy of their Cellax-DTX nanoparticles against bone metastatic PCa, Hoang et al. created a xenograft model of bone metastases by injecting human PC3 cells into the femurs of NOD-SCID mice. Survival times, tumor burden, tumor-associated pain, and bone density were assessed for three treatment groups: control, DTX, and Cellax-DTX nanoparticles. The mean survival time of the Cellax-DTX group (68 days) was more than twice as long as those of the DTX group (30.5 days) and control group (21 days). Tumor burden increased rapidly in the control group, which had a terminal endpoint of 24 days with a pain grade of 2.4. The DTX group had a terminal endpoint of 38 days with a pain grade of 2.3; at the same timepoint, the Cellax-DTX group had a pain grade of only 0.4. MicroCT bone density measurements revealed that the control group had significant bone loss 2 weeks after treatment. Although bone mineral loss in the DTX group and Cellax-DTX group did not differ significantly 2 weeks after treatment, by 4 weeks, the Cellax-DTX group had significantly higher bone density conservation than the DTX drug group did. This research highlights how the use of carefully engineered nanoparticles can make existing therapeutics more effective.

**Figure 3:**
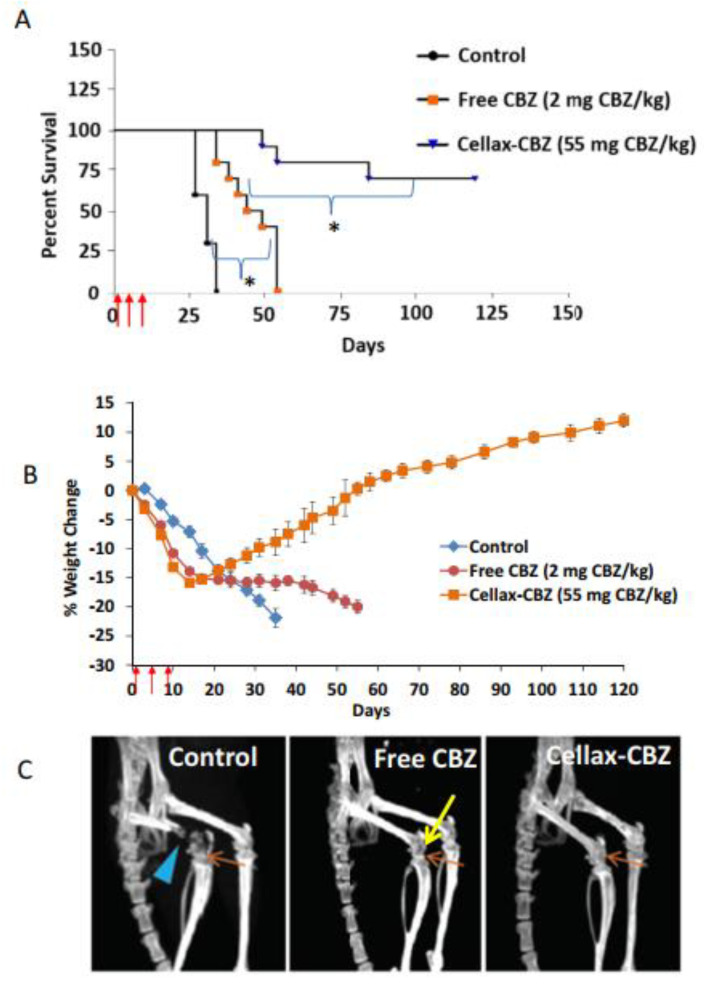
In vivo efficacy of free cabizataxel (CBZ) and Cellax-CBZ in a model of treatment-resistant bone metastatic PCa. Mice received intrafemoral injections of C4-2B-RES cells and were treated with CBZ (2 mg/kg, q159d), Cellax-CBZ (55 mg CBZ/kg, q159d), or saline control (*n* = 10 per group). Body weight loss, behavioral changes, and survival were monitored over time. Kaplan–Meier survival curves are shown in (**A**), and body weight changes are shown in (**B**). Arrows indicate injection days. * *p* < 0.05. Representative microCT images of mice from each treatment group 4 weeks after the initial dose are shown in (**C**). Orange arrows indicate tumor cell injection sites; the blue arrowhead indicates complete bone loss; and the yellow arrow indicates mild bone loss. (Adapted with permission from [159]).

In a follow-up study, Hoang et al. developed CBZ-conjugated Cellax nanoparticles for the treatment of bone metastatic PCa [159]. Currently, chemotherapy used for metastatic castration-resistant PCa starts with DTX [183]; however, drug resistance and toxicity limit its use [184]. Although CBZ is approved for DTX-resistant PCa, its effectiveness is also limited by its high toxicity and cost [185]. The authors aimed to increase the maximum tolerated dose while decreasing CBZ toxicity by using Cellax-CBZ nanoparticles as the drug delivery vehicle. DTX-resistant C4-2B-Res PCa cells were directly injected into the femoral bone marrow of NOD-SCID mice to create a bone metastatic PCa model for Cellax-CBZ evaluation. One day after implantation, the mice began treatment that consisted of three injections of saline (control), CBZ drug (2 mg/kg), or Cellax-CBZ (55 mg/kg), each delivered 4 days apart. The survival time, body weight, bone density, histology, and serology measurements for each treatment group were assessed. All mice in the control group had a constant weight loss throughout the treatment and were euthanized by day 34 (Figure 3A). The weight of the CBZ treatment group plateaued at −15% after approximately 3 weeks, but the group never recovered, and the last mouse was euthanized by day 54. The Cellax-CBZ group had a weight loss profile similar to that of the CBZ group until day 14, however the Cellax-CBZ group fully recovered the lost weight after 6–7 weeks (Figure 3B). In addition, the Cellax-CBZ treatment group had a survival rate of approximately 70% at the end of the study (120 days). After 4 weeks, microCT of the tumor implantation sites revealed fractures and significant bone loss in the control group, mild bone loss in the CBZ drug group, and healthy bone in the Cellax-CBZ group (Figure 3C). The CBZ and Cellax-CBZ groups exhibited temporary neutropenia, which returned to normal 1 week after the final treatment. For both groups, histology showed no tissue toxicity in the liver, where Cellax-CBZ nanoparticle uptake was the highest, as the liver is a common clearance route for nanoparticles [186,187].

Dozono et al. used a polymer-drug conjugate to treat a human patient with hormone-refractory PCa with multiple metastases in the lung, pelvis, and femur [160]. The polymeric portion of this drug consisted of the copolymer of (N-2-hydroxypropyl)methacrylamide (HPMA) and methacrylaminohexanoylhydrazide. Polymers of HPMA are water-soluble and drug conjugates containing these polymers can increase the solubility of lipophilic drugs and increase their half-life in circulation [188]. The drug pirarubicin (THP) was conjugated to the HPMA polymer through the hydrazide functional groups of the co-monomer to form a hydrazone bond. Hydrazone bonds are pH-sensitive; while they are stable in normal tissue, which has a pH of 7.4, they are cleaved under mildly acidic conditions like those of a tumor, which has a pH near 6.0, releasing the drug [189]. This HPMA-pirarubicin drug conjugate (P-THP) accumulates in primary tumors through the enhanced permeability and retention effect [190,191] and can accumulate in metastatic tumors [190]. The authors found that a combination therapy that included P-THP was able to significantly inhibit bone metastatic PCa growth in a patient in whom LH-RH agonist and endocrine therapy failed. The patient was given multimodal treatment, which included proton beam radiotherapy for the primary tumor and intravenous P-THP infusions (30 mg, 50 mg, or 75 mg of free THP/70 kg) every 2–3 weeks for the metastatic lesions. Pre-treatment bone scintigraphy revealed metastatic lesions in the pelvis, sacrum, and femur, and CT revealed tumor nodules in the lungs. Tumor nodules in the lungs disappeared after 2 weeks of P-THP infusions, and the bone metastases completely regressed after 20 months of treatment. Although it included only one patient, this study highlights how multimodal therapies and nanomedicine can improve the quality of life and survival of patients with bone metastatic PCa.

D. 3D-Printed Scaffolds

One of the main applications of 3-D printing since its inception has been rapid prototyping. In recent years, this advanced manufacturing technique is expanding into new areas such as biomedical devices [192]. This is mainly due to the precision that can be achieved by 3-D printers on the market today and the growing list of biocompatible materials that objects can be made from. Using a technique known as 3D-bioprinting for example, tissue engineering is achieved by 3D-printing a scaffold that mimics the extracellular matrix (ECM), while simultaneously incorporating the living cells and biochemicals needed for tissue/organ growth [193]. Of course, researchers have also demonstrated other types of implantable 3D-printed devices. Some are intended to be permanent, biocompatible devices that utilize the precision manufacturing of 3D-printing [194]. Other proposed devices are biodegradable, being naturally absorbed by the body over time after the device has served its purpose [195]. Here we discuss an example of a drug-eluting 3-D printed object developed by Ahangar et al. to locally treat PCa bone metastases [161].

In a proof-of-concept study, they explored 3D-printed scaffolds (0.6-mm-thick disks with 5-mm diameters) capable of a sustained release of doxorubicin (DOX), a potent chemotherapeutic agent. Over 7 days, the scaffolds released between 60–80% of their total DOX content, depending on the porosity of the scaffold, which the authors demonstrated was tunable depending on the type of 3D-printing filament used. In vitro experiments were performed with the LAPC4 cell line and patient-derived cells resected from a metastatic spine tumor secondary to PCa. A 7-day Alamar Blue assay revealed that, compared with control scaffolds, scaffolds loaded with 50 ng of DOX reduced the metabolic activity of both cell lines more than 65%, which is consistent with the EC50 value of DOX (0.01 uM) in LAPC4 cells. The authors acknowledged that the scaffold material does not have enough mechanical strength to serve as a bone substitute, but they suggested that their scaffolds could be reinforced with an appropriate material to create a composite medical device [196].

E. Nanodelivery platforms for gene therapy

To expedite the development and review of new drugs, the U.S. Food and Drug Administration Safety and Innovation Act of 2012 created the “breakthrough therapy” designation for certain drug applications. For a drug to receive this designation, it must treat a serious or life-threatening disease, and there must be preliminary clinical evidence that the drug demonstrates substantial improvement over existing therapies on one or more study endpoints [197]. Each year since 2014, the Center for Biologics Evaluation and Research has reviewed more than 20 biologics (i.e., drugs classified as being biological in origin) and has granted breakthrough therapy status to eight on average. Given these successes, some discussion of the recent preclinical research of biologics for the treatment of bone metastatic PCa is warranted.

In PCa patients, low expression levels of prostrate transmembrane protein androgen induced 1 (PMEPA1) have been linked to a higher incidence of bone metastasis [198,199]. To evaluate the merits of regulating PMEPA1 to elicit a therapeutic benefit in PCa, Gu et al. designed a nanoparticle delivery system for pPMEPA1 plasmid DNA [200]. First, they covalently tethered the polypeptides R7D6 (CRRRRRRRCDDDDDD; CRD) and T7 (HAIYPRH) to the N-hydroxysuccinimide and maleimide functional groups of an α-maleimide-ω-N-hydroxysuccinimide-PEG polymer, respectively. The CRD peptide contains aspartic acid residues for targeting the bone microenvironment [201], whereas the T7 peptide sequence can form a complex with the plasmid DNA pPMEPA1, forming spherical nanoparticles (CRD-PEG-T7/pPMEPA1 nanoparticles) capable of delivering genes to cancer cells [202]. Gu et al. created a human xenograft bone metastatic PCa model by injecting LNCaP cells into the tibias of 4-week-old male BALB/c nude mice. The mice were given five injections (each with an equivalent dose of 50 µg/kg pPMEPA1) over 11 days. Compared with that of the control mice, the median survival time of the mice treated with CRD-PEG-T7/pPMEPA1 nanoparticles was extended by 3 weeks, and both tumor volume and mass were significantly reduced. Although histological analysis revealed signs of toxicity in the tumors of the mice treated with CRD-PEG-T7/pPMEPA1 nanoparticles, no such signs were found in the hearts, livers, spleens, lungs, or kidneys of these mice.

**Figure 4:**
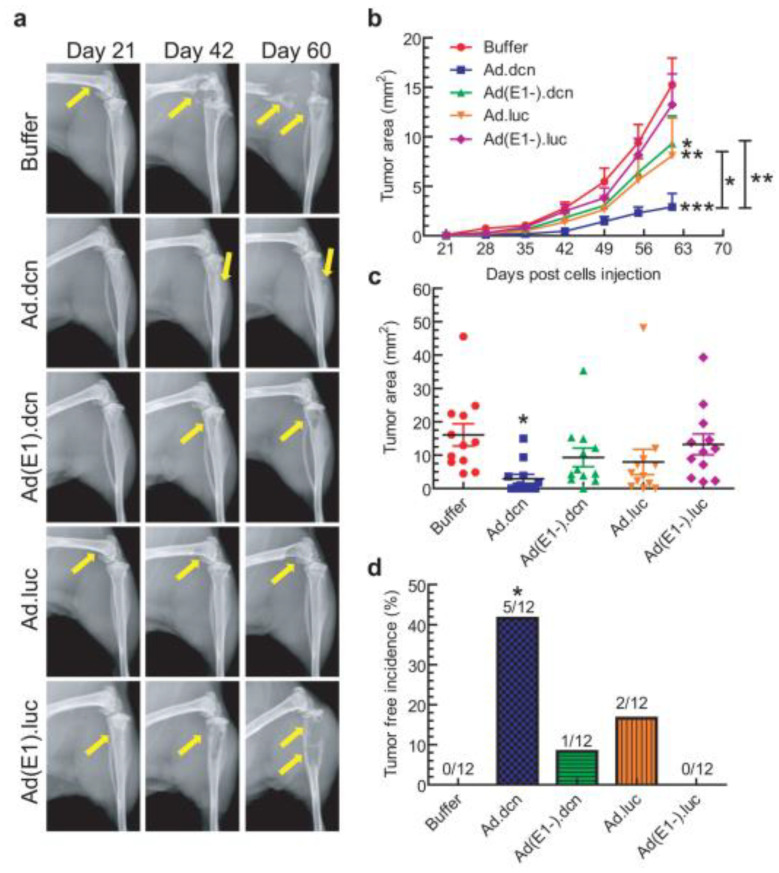
Effect of adenoviral vectors on skeletal tumor progression. (**a**) Representative radiographs of the hind limbs of mice from each treatment group on days 21, 42, and 60 are shown (*n* = 12 mice per group). Yellow arrows indicate the sites of osteolytic lesions. (**b**) Skeletal tumor progression was monitored by quantifying the radiographical sizes of lesions in both hind limb bones for the duration of the study. (**c**) Lesion sizes in the hind limb bones on day 60. (**d**) Each treatment group’s incidence of bone metastasis-free mice (i.e., mice without radiographically evident lesions) on day 60. *p* values for comparisons with the buffer group are shown in (**b**–**d**), and p values for comparisons between Ad.dcn and Ad.luc and between Ad.dcn and Ad(E1-).dcn are shown in (**b**). * *p* < 0.05; ** *p* < 0.01; *** *p* < 0.001. (Adapted with permission from [163]).

Decorin (DCN), a leucine-rich proteoglycan of 90–140 kDa, binds to type I collagen [203]. By interacting with vascular endothelial growth factor receptor 2, DCN regulates autophagy processes and can inhibit tumor angiogenesis [204,205]. DCN can also target bone metastasis microenvironments, where it inhibits osteoclastogenesis while promoting osteoblastogenesis [206,207]. To determine whether *DCN* gene therapy could effectively treat bone metastatic PCa, Xu et al. developed a recombinant adenovirus that carries the human *DCN* gene [163]. They found this adenovirus (Ad.dcn) to have dose-dependent cytotoxicity in the human PCa cell lines PC3 and DU145. Using qPCR (quantitative polymerase chain reaction) measurements of known DCN-regulated genes, such as *MET*, *CTNNB1*, and *VEGFA*, the authors also found that the DCN expressed by Ad.dcn was biologically active in infected PC3 cells. They then created bone metastatic PCa animal models by injecting PC3-luc cells into the left heart ventricles of male nude mice (Nu/Nu). Treatments of 2.5 × 10^10^ virus particles per mouse were given on days 10, 13, and 16. Radiographic measurements (Figure 4a) revealed that the untreated mice developed osteolytic lesions in their hind limbs by day 21, with significant bone loss and fractures occurring by day 60. By day 51, the Ad.dcn treatment group showed significant reduction in bioluminescent signal intensity compared to the control group (*p* < 0.001), indicating an inhibition of tumor growth which was confirmed by tumor size measurements throughout the course of the study (Figure 4b,c). In the Ad.dcn-treated group, five mice had no skeletal-related tumors (Figure 4d), and the remaining seven mice had significantly less bone loss than the untreated group did.

Atelocollagen (ATE) is a derivative of type I collagen in which the N- and C- terminal telopeptide units have been removed by pepsin treatment [208,209]. Hao et al. developed a gene regulation therapy for bone metastatic PCa that used an ATE-based system to deliver microRNA [157]. The ATE nanoparticles contained an RNA aptamer (APT) capable of binding prostate-specific membrane antigen (PSMA), increasing their specificity for PCa cells that overexpress PSMA [210,211]. The microRNAs miR-15a and miR-16-1, which act as tumor suppressors in PCa, were chosen for the gene therapy [212,213]. Assays with PC3 and LNCaP cells confirmed that the ATE-APT nanoparticles selectively bound to and were internalized by PSMA-expressing cells. Also, ATE was able to significantly slow the degradation of miR-15a and miR-16-1 in the presence of ribonuclease. The authors created a bone metastatic PCa model by injecting PCa cells into the tibias of BALB/c mice. Treatments included saline (control), miRNA–ATE–APT (negative control), an miRNA–ATE complex, and the miRNA–ATE–APT nanoparticle. Kaplan–Meier survival analysis showed that the mice in the control and negative control treatment groups had approximately equivalent survival times (27 and 28 days, respectively). Mice treated with the miRNA–ATE complex, which did not have the PCa targeting aptamer, had a mean survival time of 38 days, and mice treated with the miRNA-ATE-APT nanoparticle had a mean survival time of 57 days. Given its targeting ability, the aptamer combined with the ATE scaffold, which prevents the degradation of the miRNA, may prove a promising strategy for gene regulation strategies aimed at treating bone metastatic PCa.

Another commonly used biodegradable polysaccharide studied in many areas of biomedical research is chitosan, which is the partially de-acetylated form of chitin, a substance found in the exoskeletons of crustaceans [214]. Because chitosan contains an amine functional group, its physical and chemical properties can be altered by its derivatization with other molecules [215,216,217]. Guar et al. used the ionic gelation method to synthesize chitosan nanoparticles that could carry miRNA-34a to the bone and transfect PCa cells [156]. Previous studies had shown that miRNA-34a inhibited the growth of PCa xenografts in immunodeficient mice [218,219], and clinical data showed that miRNA-34a expression is inversely proportional to increasing PCa grade and stage [156]. The authors found that miRNA-34a overexpression, by downregulating targets such as MET, Axl, and c-Myc, induces a non-canonical form of autophagy. This autophagy, together with miRNA-34a-induced apoptosis, inhibited the proliferation and promoted the death of PCa cells with high metastatic potential.

The tripartite motif-containing protein 24 (TRIM24), which activates PI3K/AKT signaling pathways [220], is overexpressed in castration-resistant PCa relative to primary PCa [221,222]. Thus, *TRIM24* gene therapy may be beneficial to bone metastatic PCa patients. Shi et al. developed a TRIM24 delivery system using the human antibody that binds to PSMA. Using known bioconjugation chemistry, the PSMA antibody was first covalently tethered to protamine sulfate, and the modified antibody (PSP) was then able to efficiently encapsulate the TRIM24 siRNA (PSP-TRIM24) [223]. Cell assays revealed that the PSP-TRIM24 complexes were internalized by PSMA-positive cell lines (C4-2, LNCaP, PC3-PSMA+) but did not bind to or deliver TRIM 24 to PSMA-negative cell lines (DU-145, PC3). Western blot confirmed that in PC3-PSMA+ cells, TRIM24 expression was significantly decreased by PSP-TRIM24 treatment. In a bone metastatic PCa mouse model created with intratibial injections of PC3-luc-PSMA+ cells, the PSP-TRIM24 treatment significantly inhibited tumor growth and prevented bone loss compared to the control group (*p* < 0.01). Since PSMA is universally expressed in PCa metastases, this strategy has the potential to be highly specific and clinically relevant to a large patient population.

## 3. Future Outlook

The osteoblastic nature of PCa bone metastasis has been an underutilized characteristic in terms of preclinical investigations. The lack of suitable models for preclinical research limits the pace of research into PCa bone metastasis both in vitro and in vivo. Most researches use PCa cancer cell lines injected via intraosseous or intracardiac routes. However, cells developed from the PC3 cell line form osteolytic bone metastases after being injected through intracardiac, tail vein intravenous, or intraosseous routes, which are not entirely reflective of the PCa in humans. Similarly, DU145 and LNCaP cell lines produce osteolytic and mixed osteoblastic-osteolytic lesions, respectively, when inoculated in immunodeficient mice when injected intraosseously or intracardially. This has implications for the translatability of findings made using commonly used PCa cell lines. Nevertheless, the PC3 cell line remains one of the most commonly used owing to its relative aggressiveness, which refers to the ability of PC3 cells to grow easily in vivo.

An emerging area of research involves using bone microenvironment factor to recapitulate the unique osteogenic phenomenon in an experimental setting. In a study by Lin et al., overexpression of BMP4 non-osteogenic C4-2b PCa cells induced ectopic bone formation when implanted subcutaneously [47]. Meanwhile, intentionally designed nanomaterial-based scaffolds may be provisioned with favorable properties that mimic the intricate microenvironment necessary for bone formation in metastatic PCa. Past applications of a nanomaterial scaffold have shown suitable application in the induction of ectopic bone formation. He et al. observed that the in vivo implantation of a nano-hydroxyapatite-chitosan scaffold seeded with osteo-induced bone marrow mesenchymal cells resulted in greater de novo bone, collagen formation, and scaffold degradation compared to similarly implanted cell-free scaffolds. Using a similar approach, porous scaffolds may be designed to hold PCa cells with enhanced osteogenic potential to create a three-dimensional lattice for cellular seeding in the bone matrix. Additionally, the nanocomposites for the scaffold may be formulated to release growth factors or related agents that simulate the pro-osteogenic microenvironment in bone metastatic PCa tumors. A similar application has been demonstrated by Bock et al. as described previously. Such composites may be implanted into animal tissue subcutaneously for in vivo investigation or may be reconfigured to better suit in vitro experimentation. Using nanotechnology to create improved in vivo tumors to study may accelerate the pace at which safer and more effective treatments for PCa metastases are developed.

Furthermore, use of osteoblastic markers for targeted diagnostic and therapeutic interventions may be a novel approach to bone metastatic PCa. Clinical studies on bone-homing pharmaceuticals such as radium-223 and atrasentan demonstrated inhibition of abnormal bone formation as well as cancer progression [28,29,30,31,32]. Possible osteoblastic markers that may be utilized include: (1) procollagen I amino-terminal and carboxy terminal which are the propeptides for Type 1 collagen which constitutes around 90% of the bone matrix; (2) bone-specific alkaline phosphatase, a marker for middle stage bone formation; (3) osteocalcin, a noncollagenous marker of late bone formation; and (4) osteoprotegerin, produced by osteoblasts for inhibition of bone resorption [224,225,226,227]. These biomarkers have been implicated in PCa bone metastases in which nanomedicine can be used to deliver imaging and therapeutic agents specifically to the bone metastases.

Most of the nanoparticle-based treatments are still in preclinical phase, indicating challenges in translating the technology from bench to bedside. The assessment of the inherent risks of nanoparticles, such as toxicity, long-term exposure and clearance, routes of administration, as well as interaction with other biological molecules and their long-term effects, are needed for clinical translation [228]. In line with this, FDA has established the Nanotechnology Characterization Laboratory (NCL) in collaboration with the National Institute of Standards and Technology (NIST) and the National Cancer Institute which provides infrastructure to accelerate the preclinical testing of nanoparticle efficacy and toxicity to advance basic science research into the clinic.

## 4. Conclusions

Despite much effort, bone metastatic PCa is still considered incurable, as the 5-year survival rate remains very low. Thus, more effort is being focused to overcome the challenges in treating bone metastatic PCa. Advances in diagnostic imaging through multimodal imaging made possible by nanotechnology are being made that can lead to earlier interventions and better treatment response tracking. In addition, novel, more accurate translational in vitro and in vivo models are being developed to elucidate the cellular mechanisms of bone metastatic PCa and evaluate new therapeutics for the disease. Researchers are also developing a variety of nanoparticles to target and/or localize within the bone, increase the efficacy of a wide range of therapies, and decrease the toxicity of established chemotherapy drugs used for bone metastatic PCa. In preclinical research, biologics are being increasingly incorporated into experimental bone metastatic PCa treatments, which indicates that future clinical treatments for the disease could include more personalized gene therapies based on specific biomarkers present in individual patients’ cancers.

## Figures and Tables

**Table 2:** Drug delivery platforms used for bone metastatic PCa treatments.

Drug Delivery Vehicle	Material	Therapeutic Agent	Results	Refs
Polymeric Nanoparticles	Poly(d,l-lactide-co-glycolide)	Paclitaxel	~50% lower tumor burden at 5 weeks post-treatment compared to saline (*p* < 0.05). No bone loss compared to >50% bone resorption in control.	[154]
		Docetaxel	Median survival for untreated group = 10 weeks vs. >48 weeks for the treated group, which is beyond the study end point	[155]
		Cabizataxel	Significant tumor weight reduction compared to the free Cabizataxel treatment group (*p* < 0.05)	[149]
	Chitosan	miRNA	Successfully delivered tumor suppressive microRNA to PCa cells	[156]
	Atellocollagen	miRNA	PSMA targeting atellocollagen enhanced transfection efficiency of microRNA in both in vitro/in vivo models	[157]
Polymer-Drug Conjugates	Pegylated carboxymethyl cellulose (Cellax)	Docetaxel	Mean survival times 2X that of free docetaxel treated groups (*p* < 0.05)	[158]
		Cabizataxel	Approximately 4X increase in overall survival time compared to saline and free cabizataxel treatment groups	[159]
	HPMA	Pirarubicin	A single human case study reported complete tumor regression for this treatment in combination with proton beam radiotherapy	[160]
3D-printed scaffold	Poly(urethane)/poly(vinyl alcohol) copolymer	Doxorubicin	Exhibits sustained drug release (>7 days) and reduced proliferation of LAPC4 cells in vitro	[161]
Liposome	Not published	Vicrostatin	Decreased metastatic potential and inhibited tumor growth in androgen dependent in vivo model	[162]
	DSPE-PEG-2000/DPPC/cholesterol	Dexamethasone	Passively targeted bone lesions and significantly inhibited growth up to 26 days compared to empty vehicle (*p* < 0.001)	[147]
	Tocopherol acetate/Labrasol	Etoposide and Curcumin	Confirmed intracellular delivery to PC3 cells and 1.5-fold enhancement in cytotoxicity compared to free drug (*p* < 0.05)	[148]
Viral Vector	Recombinant oncolytic adenovirus	Decorin gene	Decorin expression inhibited tumor cell migration and significantly reduced skeletal metastases (*p* < 0.05)	[163]

Abbreviations: HPMA, N-2-hydroxypropyl)methacrylamide; DSPE, distearoyl-phosphatidylethanolamine; PEG, poly(ethylene glycol); DPPC, dipalmitoylphosphatidylcholine; DOPA, Dioleoyl phosphatidic acid; RGD, arginine-glycine-aspartic acid tripeptide.

## Data Availability

No new data were created or analyzed in this study. Data sharing is not applicable to this article.
